# Personal Narrative under Nationalism: Chinese COVID-19 Vaccination Expressions on Douyin

**DOI:** 10.3390/ijerph191912553

**Published:** 2022-10-01

**Authors:** Zheng Yang, Xi Luo, Hepeng Jia, Yu Xie, Ruifen Zhang

**Affiliations:** 1 Center for Chinese Urbanization Studies, School of Communication, Soochow University, Suzhou 215123, China; 2 School of Communication, Soochow University, Suzhou 215123, China

**Keywords:** COVID-19 vaccination, narration, individualism–nationalism, memetics, imitation public, Douyin

## Abstract

Scholars are divided over whether narrative/storytelling occupies a central position in health-related behaviour or in the health-related issues discussed on social media platforms. This study explored Chinese COVID-19 vaccination expressions on Douyin, China’s biggest short-video sharing social media platform, and found that narration is still the most important tool employed by Chinese users when talking about COVID-19 vaccinations on Douyin, emphasizing nationalism and widespread optimism. Most of the narratives employed by Chinese users come from a first-person perspective. Nationalism, as manifested in the support expressed for national policies, rather than the external platform characteristics of memetics, makes the Chinese users’ expressions about COVID-19 vaccinations similar on Douyin. Douyin seems to have become a ‘pilgrimage platform’ for the Chinese public to express their patriotic sentiment and their trust in the country and the government.

## 1. Introduction

The COVID-19 pandemic has led to more than 420 million infections and 4.9 million deaths worldwide, as of October 2021 [1]. To tackle the pandemic, many countries began to encourage or even require their citizens to get vaccinated for COVID-19 [2]. In China, by the end of 2020, the inactivated COVID-19 vaccine, developed by China National Pharmaceutical Group Corporation, was approved by the National Medical Products Administration of China. By October 2021, the number of COVID-19-vaccinated people in China exceeded 1.1 billion, which was nearly 80% of all Chinese citizens. Vaccination has become the most effective health initiative in response to COVID-19 and has been adopted by both the Chinese Government and the public [3,4].

While the Chinese public has been widely vaccinated against COVID-19, they have also shared their vaccination experiences and emotions on social media platforms [5]. From the beginning of the COVID-19 outbreak, compulsory lockdowns and restricted travel led to an increase in the Chinese public’s social media usage [6,7]. Social media platforms have been found to be popular for discussing various health topics and have thrived as ways for people to connect by seeking, producing, and sharing health content [8,9]. Among the different, widely used social media platforms in China, short-video-sharing platforms such as Douyin have acted as a common source for the Chinese public’s health information and experience sharing, and also as important tools for understanding the Chinese public’s opinions of these public health issues [7,9]. Previous studies on the COVID-19 vaccine discussion on Chinese social media platforms have mainly focused on Weibo and WeChat [3,10,11,12,13]. Static textual, relational, and image data make an analysis of these two platforms easier to implement. There is a relative lack of research on short-video-sharing platforms and COVID-19 vaccine video content on these platforms, a shortfall that this study intended to address. This is the first contribution of this study.

When understanding health experience-sharing on social media platforms, narrative is one of the most common analytical frameworks, turning health and illness into a visible experience [14,15]. However, recently, scholars have expressed different or opposing understandings of health-related narratives on social media platforms. Many scholars still believe that storytelling is the most common means for users to share health-related behaviour and to participate in health topic discussions [15,16,17,18]; however, some recent studies show that storytelling or narrative is not all that matters in health discussions on social media platforms [8]. It is still unclear whether storytelling/narrative plays a primary role in digital health expression. By analysing personal COVID-19 vaccine content on Douyin, this study investigated how and to what extent Chinese Douyin users rely on narratives to share vaccination information and their experiences on this short-video-sharing platform, which can contribute answers to this question in the Chinese context.

In addition to narrative, individualism–collectivism/nationalism is also an important theoretical framework for understanding users’ behaviour on social media platforms [4,19]. Some studies have emphasized the influence of nationalism on personal online health expressions and other behaviours [4,20], while others have focused on the function of social media platforms to empower individuals through a discourse space and the power to show their own experiences and characteristics [21]. However, studies of the COVID-19 vaccinations in China have found that collectivism/nationalism is an important factor affecting the Chinese public’s vaccination behaviour [4,22]. When vaccination behaviour that is influenced by collectivism/nationalism was manifested on social media platforms, such as Douyin, posted by people with clear, individualistic characters, this study investigated which side was more prominent through a quantitative content analysis of 2000 random short videos about vaccines, collected from Douyin. The findings showed that narratives and nationalism were prominent in the Chinese people’s online comments in relation to the COVID-19 vaccine. Douyin seems to have become a ‘pilgrimage platform’ for the Chinese public to express their patriotic sentiment and their trust in the country and the government. Although the content published on Douyin and other Chinese digital platforms is strictly censored by the Chinese Government, the content uploaded by individual users is still considered as a manifestation of their attitude towards a specific issue, such as COVID-19, which is also adopted as the object by many studies [5,7].

## 2. Narratives about Health-Related Behaviour on Social Media Platforms

Narrative, sometimes called storytelling, has generally been put at the centre of individuals’ conversations about health-related issues [23], and has also long been used as a framework for understanding how individuals share and express their views on health and health-related behaviours [24,25,26]. Especially the social media platforms, expanding individuals’ ability to present their own stories to others and to develop digital narratives, including health-related stories, empowers them [27]; therefore, narrative has become the basic paradigm for understanding health communication and personal health-related behaviour [16,17,18]. The functions of such narratives in health-related expressions on social media platforms are to express personal experiences and are considered to help frame the authors’ personal identity and lay expertise by emphasizing the individual differences between their stories [24]. Therefore, such narratives about health-related behaviour on social media platforms are mostly narrated from the first-person perspective [18,28].

However, a paper published in *New Media and Society* by Vicari [8] found that “non-narrative content is actually more common than storytelling in Twitter conversations”, about a specific health issue: BRCA (breast cancer gene). Through detailed empirical research, Vicari found that the sharing of professional information via different intertextualities was much more significant than personal stories of health-related behaviour on Twitter. This study overturned the long-held understanding that narrative is the centre of conversations about health-related issues, and some of the understanding about narrative being the most important textual tool in social media content sharing—for example, Twitter was directly defined as a “storytelling medium” by Papacharissi [29]. Vicari further pointed out that even in the limited narrative content about BRCA in Twitter conversations, “it is more often based on third-person narrations than on ‘self-stories’” [25]. Vicari’s findings urged a rethink on the status and role of narration in health-related behaviour on social media platforms.

From 2020, the COVID-19 pandemic caused health conversations and health-related behaviour sharing to grow rapidly on social media platforms [30]. However, the research to date on the status and role of narratives about health-related behaviour has mostly focused on Western social media platforms, such as Twitter and Facebook, with an aim to prove the centrality of narrative in health-centred threads, which can develop over time on these platforms [8,31,32,33]. Less attention has been paid to the situation in Eastern cultural environments such as China. As a genre, the functions and status of narrative are considered to change with the different contexts [34]. Different cultural environments and different digital platforms have varying approaches to narrative [8,35]. Therefore, further investigation is needed to understand narratives about health behaviours on social media in different cultural environments and on different digital platforms. Most of the current research on narratives about health-related behaviour on social media platforms focuses on static text and picture content [8,32,33], and ignores the recent rapid rise of short-video-sharing platforms, such as Douyin. Different social media dynamics could directly affect different content production, including the use of narrative [36]. Therefore, we cannot directly ‘transplant’ research results from Twitter and other platforms, which focus on static textual content, onto short-video-sharing platforms, especially given the non-Western context. Thus, the following research question was proposed:

RQ1. What role do narratives play in conversations about COVID-19 vaccinations on Douyin?

## 3. Individualism/Nationalism and Health-Related Behaviour on Social Media Platforms

With the emphasis on narrative, another important feature of health-related discussions on social media platforms is individualism, or so-called personalization [37,38]. Individualism–nationalism has also been found to be closely integrated with narrative in understanding and responding to COVID-19 in the Chinese context—some scholars refer to a “nationalist narrative” in terms of COVID-19 [39].

Nationalism means advocacy of, or support for the political independence of a particular nation or people [40]. It has always been related to patriotism in the Chinese context [41,42] and is characterized by support for China’s ruling party, *the Communist Party of China*, and its policies [43,44]. Nationalist expressions among Chinese citizens have been found both offline and online, which has been defined as ‘Chinese digital nationalism’, and includes anti-Japanese expression, support for policies, and praise for political achievements [45]. However, in the developing digital environment, since its inception, social media has been regarded as an important ecosystem, with individualistic characteristics and a power to promote personalization. It enables individual users to more freely express personal stories and emotions that are difficult to express in the traditional media environment, and to, thus, obtain more followers [21]. Wellman [46] called this phenomenon “networked individualism”: everyone is the centre of their own pluralistic social network, individuals have greater autonomy and choice, and individualism is more prominent. It is individualism rather than group, local or even national solidarities that have been reinforced by the Internet and digital networks [19]. More specifically, computer-mediated communications, developing towards personalization and ensuring interactions, are more tailored to individual preferences and needs, furthering a more individualized way of interacting and a way of mobilizing fluid networks of partial commitment [19].

In the context of networked individualism, scholars have also found that in discussions of health issues and health-related behaviour, users tend to express their personal perspective and tell stories in the first person, in an individualized way on social media platforms [47,48,49]. They use the characteristics of narrative employed in health-issue discussions and health-related behaviour, mentioned above, although Vicari’s empirical research suggested that this may not always be the case [8]. Although many studies have emphasized the important role of an online mutual aid community for patients or users to find support [50,51,52,53,54], some studies have pointed out that sympathy and empathy for other people’s illness experiences mostly derives from an individualist perspective, and there is much discussion about health and illness experiences on such online communities and social media platforms [55]. Some scholars believe that the so-called online mutual aid community is loosely made up of a group of personalized users [46]. The final construction of such an individualistic network is also a group of users that are characterised by individualism, dispersion, and fragmentation. 

Similar to the research on the use of narrative on social media platforms, discussed above, research on individualism on social media platforms has also been mostly concerned with the Western context [46,47,48,49,56,57]. However, as a concept of cultural sociology, individualism has been deeply influenced by different cultural environments [58,59]. For instance, compared with Eastern culture, Western culture is considered to emphasize and support individualism [60]. Hofstede [61] also found that there were significant differences in individualism–collectivism indicators in different countries or cultural environments. According to Hofstede Insight [62], which was based on a large-scale global survey, China’s individualism index is only 20, which is significantly lower than the main Western countries, such as the UK (89), US (91), France (71), and Germany (67)—the higher the number, the clearer the individualistic tendency of the public in that country. This reminds us that the individualism that is popular on Western social media platforms may not be significant in a discussion of health issues on Chinese social media platforms. It should be clarified that, although individualism and nationalism are highly politicalized concepts, they are, indeed, widely used and academically discussed in understanding health-related behaviour, both in the Western and Chinese context. In the Chinese cultural environment, collectivism, which corresponds to individualism, is often more narrowly expressed as nationalism, that is, in the people’s sense of identity and pride in the country [63]. Furthermore, Jia and Luo [4] found that, compared with individualism or the perception of personal benefits, nationalism and national benefit perception more readily affected Chinese people’s health choices during the COVID-19 pandemic. Therefore, based on the discussion above, when the macro nationalistic environment encounters social media platforms that stimulate individualism in the Chinese context, how this real situation is embedded in the COVID-19 pandemic is still unclear. Thus, a second research question was proposed, as follows: 

RQ2. In the conversation about COVID-19 vaccination-related content on Douyin, which is more significant: individualism or nationalism?

## 4. Methods

### 4.1. Data Collection

Launched in China as ‘Douyin’ in 2016 and internationally as TikTok in 2017, the platform has rapidly become one of the most used social media platforms, both in China and around the world [64]. In China, in April 2021, the number of daily active users of Douyin exceeded 600 million, becoming the most popular video-sharing social media platform in China. Since people were restricted to their homes during the pandemic, there has been a noticeable increase in the use of social media [7]. Among such platforms, Douyin consistently ranked highly as an information source for individuals to obtain news and as a platform for sharing their lives during the pandemic [7,65]. To explore Chinese COVID-19 vaccination-related content in short videos, Douyin is, therefore, the best research object.

By using gsdata, a Chinese social media data-scraping tool, using official APIs [66], and the keyword-based query ‘COVID-19 vaccine (新冠疫苗)’, 44,864 short videos published on Douyin between 10 March and 1 June 2021 were found. The returned data include information such as the title of the video; the original link; publishing author, area and time; number of views, likes, comments, and reposts; and the number of the publisher’s followers, etc. The collected data are all Chinese and cover all provincial administrative units in China. For further coding and content analysis, 2000 of the 44,864 short videos were randomly selected as samples for coding. Since this study involves some politicalized concepts, such as individualism and nationalism, the whole study, including data collection and the following coding and analysis, was subject to an academic review by the authors’ department to ensure the research was purely academic and politically irrelevant.

### 4.2. Coding Categories

To explore narrative/non-narrative and individualism/collectivism/nationalism in COVID-19 vaccination-related content on Douyin and further deepen understanding of this behaviour and its impact on the Chinese public’s motivations around COVID-19 vaccination, this study, firstly, divided the samples into two categories of narrative and non-narrative, based on manual coding. The narrative coding mainly refers to the elements of personal narrative as described in Orgad’s work [25], which was also used in Vicari’s study [8]: “The manual coding translated into reading the units, over and over again, to identify those incorporating ‘a chain of events, ordered […] along a timeline’ or ‘a framework that configures different events, actions and experiences into a plot”. All the COVID-19 short videos with these elements were coded as ‘narrative’. 

**Figure 1:**
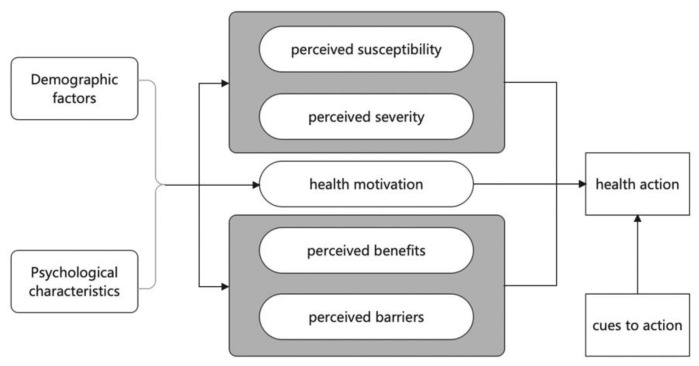
The Health Belief Model (referring to Abraham et al., 2005) [70].

The study also adopted the Health Belief Model (HBM) as its theoretical framework to analyse the individualism/nationalism content in narrative/non-narrative COVID-19 short videos. The HBM was developed in the early 1950s and has been widely used in sociology of health and illness, health communication, and related fields to understand the behaviour and motivations of people in their adoption of disease prevention strategies [67,68,69]. The HBM derives from psychological and behavioural theories with the foundation that the two components of health-related behaviour are as follows: (1) the desire to avoid illness, or, conversely, to get well if already ill; and (2) the belief that a specific health action will prevent or cure illness, involving six main parts—perceived susceptibility; perceived severity; perceived benefits; perceived barriers; self-efficacy; and cue to action (Figure 1) [70]. These six constructs, combined with some existing studies adopting health belief models and focusing on Chinese public responses to the pandemic [71,72,73], constitute the main content of the coding table. For instance, does the video and its descriptive texts, such as the title, show the consequences of the COVID-19 infection, such as symptoms, severe illness rate, or mortality rate? If yes, HBM–severity would be coded as ‘yes’, otherwise it would be ‘no’. The COVID-19 vaccination and other prevention behaviours in China have some unique characteristics, such as the government’s overall intervention and politicization tendency [74], the obvious patriotic intention of the Chinese public [4], and the vaccine certificate implemented by the government [75]. Based on these characteristics, some coding categories were added to the codebook that are specific to the Chinese context, such as ‘accessibility–country’, which means showing thanks to China for providing vaccinations, and ‘national calls’ in ‘HBM–cues to action’, demonstrating that “I was vaccinated against COVID-19 in response to the country’s call for vaccinations”, or similar content in the short videos.

### 4.3. Coding and Reliability

The coding was carried out by a team of six coders, including the first author. All six coders were trained by the second authors over five sessions until the coding feasibility of all categories was above 0.8. The intercoder reliability test was based on 50 short videos, taken from a random sample of the 2000 short videos. The reliability scores for the average pairwise Cohen’s kappa is shown in Table 1. Although a value of 0.60 is an acceptable level [76,77], in this study we raised the standard to 0.80 to ensure that the coding results were more credible, with an average reliability of 0.915. Following the intercoder reliability test, each coder was responsible for coding 330 short videos, while the first author coded 350. If the video’s link failed or the author had deleted the videos, a replacement was randomly selected from the original 44,864 pieces of data.

## 5. Results

### 5.1. More Narratives in the COVID-19 Vaccination Expressions on Douyin

Recently, scholars have demonstrated different opinions on whether narrative/storytelling is the centre of the conversation about health-related topics on social media platforms [14,16,17,18]. The coding results in this study show that narrative is still the main method used by the Chinese public to discuss or share their experiences of COVID-19 vaccinations on Douyin, especially in the following two main categories of COVID-19 vaccination short-videos on Douyin: ‘Individual (general)’ (*x*^2^ = 1189.503, *p* = 0.000) and ‘Organization (general)’ (*x*^2^ = 81.219, *p* = 0.000), responding to RQ1 (Table 2).

**Figure 2:**
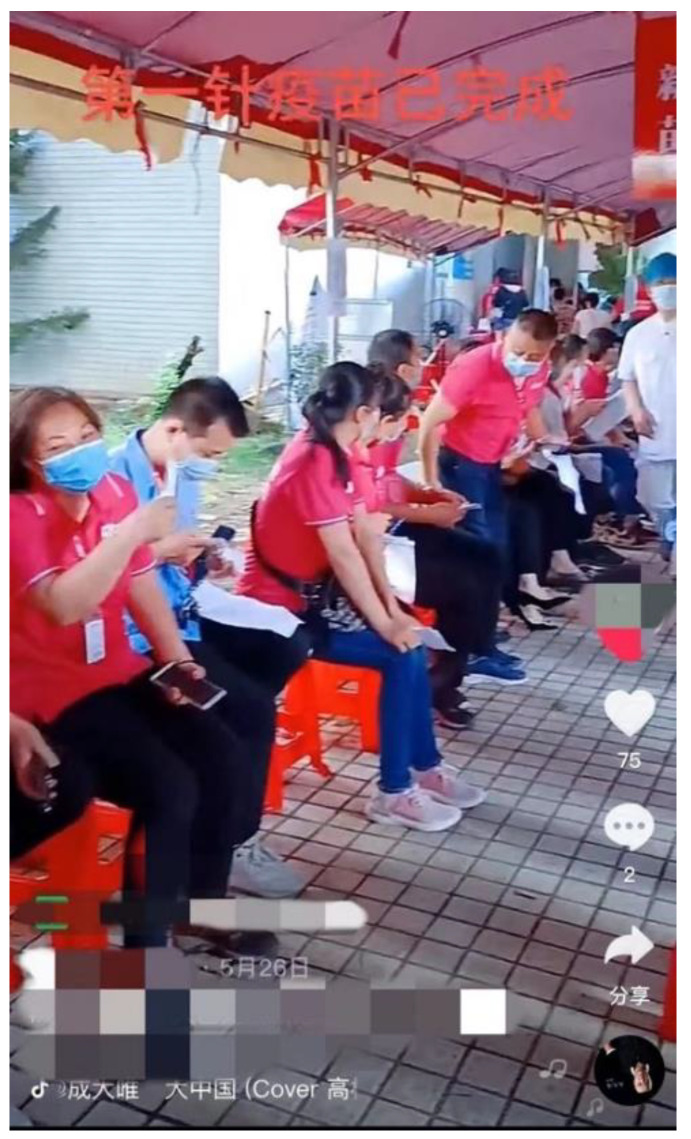
Example of the narratives (*‘the first dose of vaccine has been completed’*) used in the Chinese COVID-19 vaccination expressions on Douyin.

Among the discussions about COVID-19 vaccinations on Douyin from four different kinds of sources, the content from the ‘Individual (general)’ category accounts for most of the total sample (83.45%). Only in the ‘Individual (health-related)’ category, such as doctors, nurses, and medical experts—which occupies a very small proportion of the total sample (1.25%)—narrative does not occupy the dominant position (44%). In the other three categories, narrative occupies a dominant position. Non-narrative content, such as official news, government announcements, vaccine policies, scientific data, research conclusions, and medical suggestions only account for a very small proportion of the total. In the content published in the category ‘Individual (health-related)’, the experts are more inclined to share professional knowledge and related suggestions, such as matters needing attention after vaccination, therefore, narrative is not the main method employed. For those narratives—contrary to Vicari’s (2020) conclusion that “when story-telling does appear, it is more often based on third-person narrations than on self-stories” (p. 18)—in the COVID-19 vaccination content on Douyin, first-person narratives occupy the greatest part in both the ‘Individual (general)’ and the ‘Organization (general)’ categories, with significant differences with other narrative perspectives (*x*^2^ = 2854.612, *p* = 0.000; *x*^2^ = 174.601; *p* = 0.000) (Table 3). The Chinese Douyin users prefer to represent their own COVID-19 vaccine stories in their short video content and they use such stories to connect with the larger national pandemic environment. The coding data also showed that, in the COVID-19 vaccine stories, Chinese Douyin users tended to illustrate both the vaccination process and the results. Most short videos included the vaccination process and the results at the same time. For instance, User 1 showed the process of vaccination through a presentation of the queuing process from a first-person perspective, which displayed red onscreen characters (*第一针疫苗已完成—‘the first dose of vaccine has been completed’*) and showed the results of the vaccination (Figure 2). Such narratives about the vaccination experience were very common in the coding samples, which can be summarized under the following two narrative frames: “I’m getting vaccinating now” and “I’ve been successfully vaccinated”.

Therefore, referring back to RQ1, the answer from this study is that the Chinese public are more inclined to use narrative in their COVID-19 vaccination expressions on Douyin.

### 5.2. Emphasized Nationalism in the COVID-19 Vaccination Expressions on Douyin

**Figure 3:**
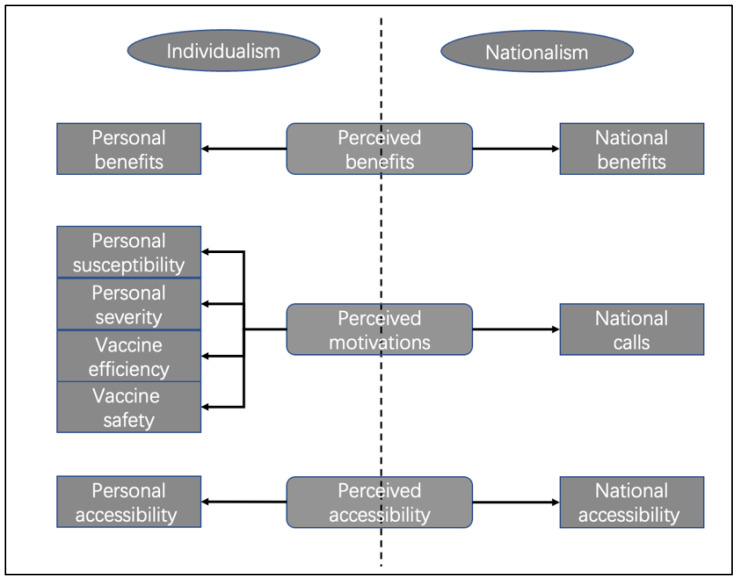
Analysis framework of individualism–nationalism in Chinese COVID-19 vaccination expressions on Douyin.

**Figure 4:**
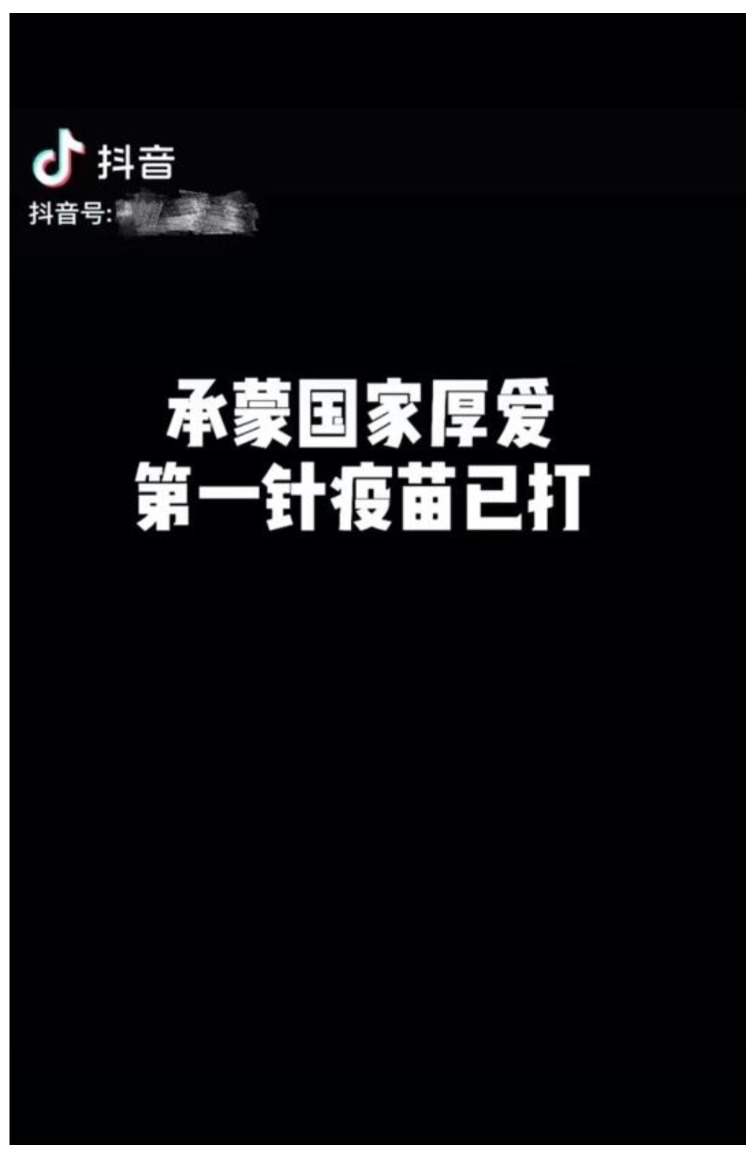
Example of national accessibility in Chinese COVID-19 vaccination expressions on Douyin (*‘Thanks to the great love of the country, the first dose of vaccine has been given*’).

As discussed above, many studies have found that the discussion of health issues and health-related behaviour on social media platforms is individualist or personalized [37,38,46,47,48,49,56,57], while recent studies in the Chinese context have found that nationalism is an important factor influencing vaccination [4,22]. To explore the situation on Chinese social media platforms and to answer RQ2, this study divided content about vaccinations on Douyin into a binary division—individualism vs. nationalism—based on the coding categories in Figure 3. Personal benefits— such as “I get vaccinated to make me better immune to COVID-19”; personal susceptibility, such as ”I feel like I am susceptible to being infected with COVID-19, so I came to get the vaccine”; personal severity, such as “getting infecting with COVID-19 is too scary for me, so I came to get the vaccine”; vaccine efficiency, such as “I feel that the vaccine is effective for my personal prevention of COVID-19”; vaccine safety, “I feel that the vaccine is safe for me without side effects”; and personal accessibility, such as “through my personal efforts, I can get the COVID-19 vaccine”—are classified as individualism-related. Correspondingly, national benefits—such as “I am vaccinated against COVID-19 for our country to achieve universal epidemic prevention as soon as possible”; national calls, such as “I am vaccinated against COVID-19 in response to the call of my country”; and national accessibility, such as “I thank the country for providing me with the COVID-19 vaccine”—are classified as nationalism-related. In all the ‘Individual (general)’ vaccine-related short videos (n = 1669), except for most of the videos that involve neither obvious individualism nor nationalism, such as those that simply present vaccine queues or vaccination results, the manifestation of nationalism significantly exceeds that of individualism, in all of the following three dimensions: personal benefits–national benefits (*x*^2^ = 71.837, *p* = 0.000); personal calls–national calls (*x*^2^ = 178.126; *p* = 0.000); and personal accessibility–national accessibility (*x*^2^ = 225.869; *p* = 0.000) (Table 4). Regarding the perception of the benefits of the vaccine, Chinese Douyin users are not inclined to say that the vaccination can help them, personally, to obtain relevant benefits but are more inclined to say that vaccination can help their motherland cope better with the COVID-19 epidemic. Among the comments, “Wish the mountains and rivers be safe (愿山河无恙)”, which means that the speaker wishes the motherland safety and success, and “Help the motherland build the Great Wall of Immunity” are the two most common expressions of national interest. Similarly, in the expression of their motivation for getting the COVID-19 vaccination, the perception dimensions in the traditional health belief model, such as perceived susceptibility, severity, and vaccine efficiency and safety, are extremely rare in the Chinese COVID-19 vaccination expressions on Douyin. However, more users clearly showed their motivation to vaccinate “in response to the call of the country”, such as National Epidemic Prevention (全民防疫)—this slogan, created by the state, has become an important part of Douyin short video titles. Finally, in terms of the availability of the vaccines, Chinese Douyin users were also more inclined to express their gratitude that “the country provides vaccines”. For instance, in one user’s short video (Figure 4), before showing her vaccination process, she used a separate picture and bold font to emphasize the following: “Thank you for the love of the motherland, the first vaccine has been delivered (承蒙祖国厚爱， 第一针疫苗已打)”, expressing her gratitude to the country for providing the free vaccines. Compared with the individualism that is considered to occupy a prominent position in the health-related behaviour on social media platforms, in the Chinese COVID-19 vaccination expressions on Douyin, nationalism is greatly emphasized. Among these expressions, Chinese Douyin users regard personal vaccination as a direct manifestation of nationalism and patriotism. This may be related to the compulsory vaccination policy implemented by China. The Chinese Government has linked the importance of the vaccination with national security, so that it is easy to establish the following sentiment: “I need to be vaccinate for my country”, in the hearts of the general public [4].

Therefore, referring back to RQ2, the answer from this study is that the Chinese public tends to express patriotism rather than individualism in their COVID-19 vaccination expressions on Douyin. 

### 5.3. Concomitant Optimism in the COVID-19 Vaccination Expressions on Douyin

**Figure 5:**
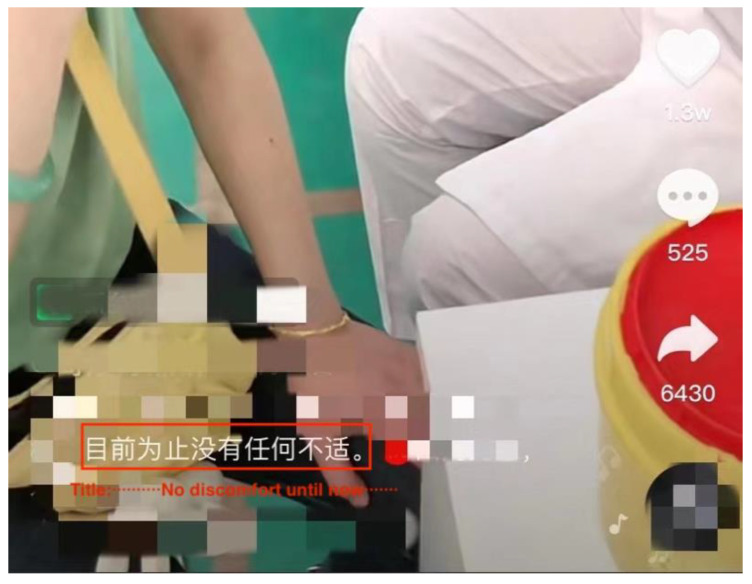
Example of refutation of side effects in Chinese COVID-19 vaccination expressions on Douyin.

Many studies have shown that stronger nationalism can enable citizens to hold a more positive attitude or optimism in the face of uncertain events [78,79]. Especially in the Chinese context, nationalism has been described as “a key component of revolutionary optimism” [80]. Trust in the country can also be transformed into optimism through vaccinations and the other anti-epidemic measures implemented by the country [4,81]. This study also found that, with an emphasis on nationalism, there is also strong optimism about vaccines in the Chinese COVID-19 vaccination expressions on Douyin. Firstly, while 1.95% (n = 39) of the videos expressed a neutral attitude towards the vaccine, the remaining 98.05% (n = 1961) clearly expressed support. In the selected samples, no videos expressed their opposition to the vaccine. This may also stem from the Chinese Government’s censorship and filtering of online content. However, more direct evidence from the perceived barriers shows that the Chinese public does have significant optimism when talking about their COVID-19 vaccination experience on Douyin. The proportion of the refutations of such barriers, such as concerns about the vaccine’s safety or the side effects of vaccination, are much higher than that of the perceived barriers, among all of the following four perceived barriers: concerns about vaccine’s safety (*x*^2^ = 106.132, *p* = 0.000); vaccine’s low effectiveness (*x*^2^ = 15.517; *p* = 0.000); side effects (*x*^2^ = 309.654; *p* = 0.000); and costs (*x*^2^ = 227.287l; *p* = 0.000) (Table 5). For instance, “No discomfort after receiving the COVID-19 vaccine” appears in the headline of many Chinese COVID-19 vaccination short videos on Douyin, to refute the possible negative physiological effects of the vaccine by employing users’ own experiences (Figure 5). In addition, in a common piece of background music used in the videos, the one sentence highlighted most often is: “COVID-19 vaccine is free”. Due to the memetic feature of the Douyin platform, users tend to be ‘imitation publics’ [64]. As a result, such background music and opinions, which refute the cost of the COVID-19 vaccinations in China, were widely copied and disseminated. This optimistic attitude towards the vaccines can be further understood as trust in China and its government in the context of its promotion of the vaccines, demonstrating Douyin users’ nationalism. Therefore, the additional finding, beyond RQ1 and RQ2, is that the Chinese public has a positive attitude towards the COVID-19 vaccinations, as presented by themselves on Douyin, which aligns with their nationalistic attitude.

## 6. Discussion

Zulli [64] observed that “scholarship in Douyin is still in its infancy”, especially as an important platform for disseminating health-related content [82]. Firstly, this study used empirical evidence to supplement the understanding of health-related expressions on Douyin, in the context of the COVID-19 pandemic. Secondly, this study responded to the discussion about narratives in health-related behaviour on social media platforms. Contrary to Vicari’s claim that “stories matter, but they are not all that matters on social media platforms” and “when storytelling does appear, it is more often based on third-person narrations than on ‘self-stories’”, this study showed that, in Chinese COVID-19 vaccination expressions on Douyin, narratives—especially those from the first-person perspective—comprised most of the overall video sample. The reason for the different research results may, firstly, relate to the socio-cultural context. Vicari mostly focused on the Western context, while this study focused on China, a country with a narrative tradition. In addition, the ideographic attributes and grammatical structure of the Chinese are also inclined to be used for narration [83,84]. Therefore, the Chinese public, who have long been immersed in this socio-cultural background and grammatical structure, may show more narrative tendencies than the Western public when facing the same event on one social media platform. Secondly, the differences in the results may also come from the differences of the different platforms. Unlike Twitter, where the content is more static text- or picture-led, although there are also a small number of videos, Douyin is solely a short-video-sharing social media platform. The video itself is more inclined to represent a story than static text, with embedded time cues [85,86]. Finally, as an event in action, the COVID-19 vaccination process has a more obvious story-like feature than the discussion of BRCA, regarding which more knowledge and experience sharing was shown in Vicari’s study. Therefore, the above-mentioned differences are a reminder that when we analyse narrative in health-related behaviour on social media platforms, we need to pay more attention to the specific platforms, issues, and macro socio-cultural differences, on a case-by-case basis. We cannot draw a conclusion about whether the narrative is greater or lesser in the health-related behaviour on social media platforms.

This study also found that in Chinese COVID-19 vaccination expressions on Douyin, compared with individualism, nationalism has been greatly emphasized by the users, with widespread optimism toward COVID-19 vaccines. Such expressions of nationalism also make the number and rate of COVID-19 vaccinations on the Douyin platform appear more than on other social media platforms in China, such as Weibo and WeChat. It seems that on Douyin, based on the expression of nationalism, the Chinese public seem to present the sentiment that it is important to receive the COVID-19 vaccination. In the context of the COVID-19 vaccination, Douyin seems to have become a ‘pilgrimage platform’ for the Chinese public to express their patriotic sentiment and trust in the country and government. Although these inner emotions are very similar and most of the users showed their own vaccination process and the results, Chinese users’ specific expressions about their COVID-19 vaccination experiences are different from those found by Zulli and Zulli’s [64] observations about the imitation and replication, based on the memetic features of Douyin. Zulli believes that there are many similar videos on Douyin because both the platform and users use mimesis as a “particularly advantageous strategy to engender content production and spread-ability in unparalleled ways”. Therefore, their similarities tend to be expressed in external ways, such as rhythm, composition, the pattern of the story, and background music, which has been called “genre” by Zulli and Zulli [65]. However, the specific content of the Chinese COVID-19 vaccination expressions on Douyin is very different. Although most users expressed similar nationalist comments and optimism with similar background music and lens features, their story content, and methods of narrating their stories varied from person-to-person. Therefore, those Chinese users cannot simply be called ‘imitation public’ because the similarity among them is not derived from the external memetic features of Douyin, but from the traditional collectivism and nationalism internalized among the Chinese public. Such spirit is more likely to be stimulated in the face of sudden national disasters. For example, during the Wenchuan earthquake in 2008, scholars discovered that a blog that had just emerged at the time became a place for Chinese netizens to spontaneously express their patriotic and collective sentiments, without a deliberate platform or government dominance [87]. Therefore, when we analyse the imitation and replication on the Douyin platform, perhaps we need to consider not only the micro platform strategy, but also the greater macro background, such as the current COVID-19 pandemic and socio-cultural contexts. In this study, rather than calling the Chinese users who appear in this study “imitation publics based on memetic genre”, I prefer to call them a nationalism-networked public, with nationalism being the concept that links them on Douyin.

In fact, on Douyin, not only do the public expressions of health issues related to the COVID-19 vaccination have an obvious tendency towards narration and nationalism, due to the platform characteristics of Douyin and the characteristics of the Chinese cultural context, some studies on the expressions of other health issues, such as sexual orientation or wearing masks, also have similar findings [88,89,90]. The important role of narrative and nationalism in the public’s expression of health issues can be found not only on the Douyin platform, but also on other Chinese social media platforms, such as Zhihu, Weibo, and WeChat [91,92,93]. Chumakov et al. (2021) also found that not only in China, but also in the Western cultural context, nationalism also contributes a lot to the public expression and acceptance of the COVID-19 vaccination [94]. Therefore, the results of this study are not just relevant to a specific case in China, but they also have a certain universal significance.

## 7. Conclusions

To conclude, this study shows that narrative is still the most important method used by Chinese users to discuss COVID-19 vaccinations on Douyin, with an emphasis on nationalism and widespread optimism. However, determining whether narrative and nationalism are as prominent in relation to other socio-cultural environments, other platforms, and other events will require further research. Furthermore, due to China’s special political pattern and the social network censorship system, where social media in China is highly controlled by the government, many personalized expressions cannot be effectively presented on digital media platforms such as Douyin. Therefore, the narratives embedded in the videos that were analysed in this study may not be representative of the authentic experiences or of the feelings of the whole Chinese nation, which is also a limitation of this study, regarding data acquisition and analysis.

## Figures and Tables

**Table 1:** Coding categories and intercoder reliability.

Coding Category	Coding Subcategory		Intercoder Reliability
Content	Information/Source		0.95
	Narrative		
Source	Individual (general)		0.98
	Organization (general)		
	Individual (health-related)		
	Organization (health-related)		
Attitude	Pro-vaccine		0.92
	Anti-vaccine		
	Neutral		
HBM–Susceptibility	No		0.99
	Yes		
HBM–Severity	No		0.99
	Yes		
HBM–Perceived Benefits	Personal benefits	No	0.83
		Yes	
	Other benefits	No	0.91
		Yes	
	Family benefits	No	0.98
		Yes	
	Community benefits	No	0.99
		Yes	
	National benefits	No	0.87
		Yes	
HBM–Perceived Barriers	Concerns about vaccine’s safety	No	0.91
		Yes	
		Refutation	
	Vaccine’s low effectiveness	No	0.94
		Yes	
		Refutation	
	Side effects	No	0.90
		Minor effects	
		Major effects	
		Refutation	
	Costs	No	0.83
		Time cost	
		Economic cost	
		Refutation	
HBM–Self-efficacy	Steps to take vaccine	No	0.87
		Yes	
	Accessibility (vaccination result)	No	0.83
		Accessibility–general	
		Accessibility–country	
		Barriers to accessibility	
HBM–Cue to action	National calls	No	0.85
		Yes	
	Expert testimony	No	0.92
		Yes	
	Personal story	No	0.92
		Yes	

**Table 2:** Narrative practices in the different categories of COVID-19 vaccination on Douyin.

Individual (General)		Organization (General)		Individual (Health-Related)		Organization (Health-Related)	
1669 (83.45%)		292 (14.6%)		25 (1.25%)		14 (0.7%)	
*x*^2^ = 966.919				*x*^2^ = 3.103			
*p* = 0.000				*p* = 0.078			
Narrative	Non-narrative	Narrative	Non-narrative	Narrative	Non-narrative	Narrative	Non-narrative
1539 (92.2%)	130 (7.8%)	223 (76.4%)	69 (23.6%)	11 (44%)	14 (56%)	12 (85.7%)	2 (14.3%)
*x*^2^ = 1189.503		*x*^2^ = 81.219		*x*^2^ = 0.360		*x*^2^ = 7.143	
*p* = 0.000		*p* = 0.000		*p* = 0.549		*p* = 0.008	

**Table 3:** Different narrative practices related to COVID-19 vaccinations on Douyin.

Individual Narrative (General) (n = 1539)				
Narrative perspective				
First-person	Second-person		Other	*x*^2^ = 2854.612
1501 (97.5%)	29 (1.9%)		9 (0.6%)	*p* = 0.000
Narrative Content				
Vaccination process		Vaccination results		*x*^2^ = 7.759
1319 (85.7%)		1466 (95.3%)		*p* = 0.005
Organization narrative (general) (n = 223)				
Narrative perspective				
First-person	Second-person		Other	*x*^2^ = 174.601
165 (74.0%)	11 (4.9%)		47 (21.1%)	*p* = 0.000
Narrative Content				
Vaccination process		Vaccination results		*x*^2^ = 71.837
201 (90.1%)		176 (78.9%)		*p* = 0.000

**Table 4:** Statistical results of individualism–nationalism among Chinese COVID-19 vaccination expressions on Douyin.

	Individualism-Related		Nationalism-Related		
Personal benefits		9.1% (152)	National benefits	20.4% (340)	*x*^2^ = 71.837 *p* = 0.000
Personal Calls 2.4% (40)	Personal susceptibility	0.1% (2)	National calls	16.6% (278)	*x*^2^ = 178.126 *p* = 0.000
	Personal severity	0.2% (3)			
	Vaccine efficiency	0.5% (8)			
	Vaccine safety	1.6% (27)			
Personal accessibility		4.7% (78)	National accessibility	24.6% (410)	*x*^2^ = 225.869 *p* = 0.000

**Table 5:** Statistical results of perceived barriers in the Chinese COVID-19 vaccination expressions on Douyin.

HBM–Perceived Barriers	Concerns about vaccine’s safety (n = 212)	Yes	14.6% (31)	*x*^2^ = 106.132 *p* = 0.000
		Refutation	85.4% (181)	
	Vaccine’s low effectiveness (n = 58)	Yes	24.1% (14)	*x*^2^ = 15.517 *p* = 0.000
		Refutation	75.9% (44)	
	Side effects (n = 347)	Minor effects	22.8% (79)	*x*^2^ = 309.654 *p* = 0.000
		Major effects	1.1% (4)	
		Refutation	76.1% (264)	
	Costs (n = 418)	Time cost	40.7% (170)	*x*^2^ = 227.287 *p* = 0.000
		Economic cost	0.2% (1)	
		Refutation	59.1% (247)	

## Data Availability

Materials and anonymous data are available from the authors upon request.
